# Is it time to recast the principles of antimicrobial prophylaxis?

**DOI:** 10.1186/1749-7922-6-21

**Published:** 2011-07-26

**Authors:** Mark A Malangoni

**Affiliations:** 1 American Board of Surgery, 1617 John F. Kennedy Blvd., Suite 860, Philadelphia, PA, 19103-1847, USA

## Abstract

Perioperative antimicrobial prophylaxis has been a time-honored principle in the prevention of surgical site infection. Its effectiveness has recently been questioned. Potential reasons for the lack of demonstrable efficacy and suggestions for re-examination of this concept are presented.

## 

The principles of perioperative antimicrobial prophylaxis were established more than 40 years ago [1]. This concept has been applied to many areas of surgery and numerous prospective randomized trials have repeatedly demonstrated that surgical site infections (SSIs) are reduced when the right antibiotics are administered appropriately. This practice has been incorporated into standardized guidelines for perioperative use through the Surgical Care Improvement Project (SCIP) and serves as a major process measurement for appropriateness of practice [2]. First and second generation cephalosporins have been the major drug class recommended and used for prophylaxis for decades and there has been little change in these recommendations over time.

Recent reports have demonstrated a lack of correlation between the use of guideline-directed perioperative antimicrobial prophylaxis, that is, administration of the right drug at the right time for the right duration and its primary outcome measure, prevention of SSI [3,4]. This begs the question: could we have been wrong about the benefits of perioperative antimicrobial prophylaxis?

There are a number of potential explanations for these observations. This principle has been so widely accepted that some propose that all patients receive antimicrobial prophylaxis regardless of the operation and risk of infection [5]. This concept fails to consider the risk: benefit ratio of even single dose drug use, since there is a small but defined risk of allergic and other adverse reactions associated with most antibiotics. Overuse blurs the advantage of prophylaxis, as many who wouldn't benefit would still receive prophylaxis and supports the concept of unrelated attribution. This is exemplified as "if you want to show the benefit of a drug, give it to those who don't need it; it works every time." Although it is recognized that perioperative prophylaxis is not the only preventive measure for SSI, failure to apply other measures such as appropriate skin cleansing, scrubbing of operating room personnel, use of aseptic technique, mechanical bowel preparation, and avoidance of undo contamination subjects patients to complications and can negate the beneficial effects of prophylaxis. In addition, the increasing prevalence of minimally invasive surgical procedures, which are associated with a lower risk of SSI than open operations for the same conditions, may also be impacting these observations [6].

We now understand that there are patient characteristics that also affect the risk of infection and can negate the beneficial effects of antimicrobial prophylaxis. These include glycemic control, tissue dessication, hypothermia, obesity, smoking, immunosuppressive drugs, nutritional state, and local tissue hypoxemia. Addressing each of these contributors requires a well-coordinated, team-based approach in order to consistently optimize the strategy to prevent SSI.

In spite of the complexity of this problem, there are other questions about perioperative prophylaxis that have not been adequately addressed. For instance, three of the most common pathogens for SSIs- *Staphylococcus aureus*, coagulase-negative staphylococci, and enterococci- are frequently resistant to currently recommended agents. Should we expect that prophylaxis that is not demonstrable in vitro will work in our patients? Patients frequently report a history of allergic reaction to beta-lactam drugs and as a result, secondary agents are used. The data for selection of these agents are often based on expert opinion rather than class 1 or class 2 evidence [7]. Is it possible that our assumptions about their effectiveness are wrong? We know that the prevention of SSI also depends on delivery of an effective concentration of antibiotic to the site at risk for infection, in this case the surgical incision. With cephalosporins, tissue concentrations are often dependent on weight-based dosing and so adjustments need to be made for overweight and obese patients [8]. Do we know the compliance with this principle?

There has been much progress made in surgery over the four decades since the benefits of perioperative antimicrobial prophylaxis were demonstrated in a prospective, randomized clinical trial. We now understand more about the complex interactions that affect SSI. We need to look to the challenges ahead and consider whether new principles need to be formulated.
